# Synthesis of Low Melting Temperature Aliphatic-Aromatic Copolyamides Derived from Novel Bio-Based Semi Aromatic Monomer

**DOI:** 10.3390/polym10070793

**Published:** 2018-07-19

**Authors:** Syang-Peng Rwei, Palraj Ranganathan, Whe-Yi Chiang, Yi-Huan Lee

**Affiliations:** Institute of Organic and Polymeric Materials, Research and Development Center for Smart Textile Technology, National Taipei University of Technology, Taipei 10608, Taiwan; rangapalraj@gmail.com (P.R.); wheyichiang@gmail.com (W.-Y.C.); yihuanlee@mail.ntut.edu.tw (Y.-H.L.)

**Keywords:** polyamide, bio-based semi aromatic polyamide salt, melt condensation process, PA6, crystallinity

## Abstract

This work investigated the synthesis of a novel low melting temperature polyamide 6 (PA6) copolyamide (PA6-BABT/SA) with different aliphatic/aromatic units weight content using a melt poly-condensation process. The bio-based aromatic *N*^1^,*N*^4^-bis(4-aminobutyl) terephthalamide diamine (BABT) and long-chain aromatic polyamide salt (BABT/SA, salt of BABT, and sebacic acid), components used for the synthesis of copolyamides, were obtained from bio-based monomers. For the first time, the pertinent BABT/SA aromatic polyamide salt was isolated as a white solid and completely characterized. By varying the weight ratio of BABT/SA salt, a series of copolyamides with different molecular weights and physical properties were prepared. The aromatic BABT/SA salt disrupted crystallization of the final copolyamides and lowered the onset of melting. The Fourier transform infrared spectroscopy and X-ray diffraction results indicated a steady decrease in the degrees of crystallinity with increasing BABT/SA salt segment ratio. Furthermore, compared to neat PA6, the obtained PA6-BABT/SA copolymers possessed a similar thermal stability and high transparency, but lower glass transition temperature around human body temperature. The PA6-BABT/SA copolymers with number-average molecular weight ≥30,000 Da presented good mechanical properties, specifically showing excellent tensile strength and elongation at break up to 105.2 MPa and 218.3%, respectively.

## 1. Introduction

Analyzing alternative bio-based renewable feedstocks to meet the constantly increasing demands of the chemical industry and is an essential step toward sustainable development. The overall aim of this study was to determine the possibility of acquiring economically feasible and sustainable polymers from renewable resources. Polyamides belong to a subclass of polymer that are generally linear and semicrystalline, which are extremely versatile engineering thermoplastics for a wide range of relevant applications in industrial fields [1]. Polyamide 6 (PA6) is one of the most widely used engineering plastics, generally prepared via ring opening polymerization (ROP) of ε-caprolactam (CPL) [2,3], which can be derived from fermented glucose [4,5,6]. PA6 is a semicrystalline thermoplastic polymer with a relatively high melting temperature (*T*_m_). Given its good mechanical strength, impact resistant, rigidity, and low density, it is broadly used in industrial applications [7,8]. Since PA6 displays excellent thermal and mechanical properties, copolymerization of CPL with numerous comonomers is of industrial and research interest because the process allows the tailoring specific properties for specific applications [9,10,11].

Comonomers, such as aliphatic or aromatic monomers, are usually used in PA6 to alter physical and mechanical properties, such as melting temperature, processability, and optical clarity [12,13,14,15,16]. Such alteration usually causes deterioration of the crystallization behavior of PA6, deformation of the crystalline phase, and undesired changes from the crystalline to amorphous phase. These changes result in a reduction in its melting temperature, crystallization rate, and the degree of crystallinity, leading to unpleasant changes in mechanical and physical properties [17]. However, low *T*_m_ copolaymides are especially suitable for hot melt adhesive applications. In addition, amorphous polymers have a lower softening temperature (*T*_s_) and improved solubility with respect to crystalline analogs, enabling their application in the fields of films, gas-separation membranes, coatings, engineering plastics, polymer blends, and composites [18,19,20]. Compared with binary polymers, copolymers usually have more interesting and comprehensive properties. Some polyamides are prepared from a combination of aliphatic and aromatic monomers. They are known as aliphatic-aromatic polyamides and typically have better solubility than aramids, and superior mechanical, thermal, and optical properties than nylons [21].

A number of attempts have been made to increase and alter the mechanical and physical properties of aliphatic polyamides by altering aromatic polyamides [22]. Novitsky et al. [23] prepared polyamide 12, T-polyamide-6-block copolymers by anionic polymerization exhibiting a blocky microstructure with good thermal and swelling properties. Following this approach, Hassani et al. modified a PA6 by simultaneous anionic ring opening polymerization and condensation reactions, with the incorporation aromatic amides into a PA6 backbone, to tune the thermal properties of PA6 [24]. However, studies of semi-aromatic polyamide salt modified PA6 copolyamides have been limited [25]. Since the design and synthesis of a bio-based polymer with a novel structure and new performance from mass-produced bio-based monomers are more valuable and important, a tremendous opportunity exists in developing classes of more sustainable plastics. Dimethyl terephthalate (DMT) is a well-recognized large-scaled bio-based monomer that can be obtained from biomass. DMT is the diester formed from terephthalic acid (TPA). In addition, TPA has a small carbon footprint, is low-cost, and can be produced in large-scaled highly optimized processes, such as a monomer for PET [26,27]. 1,4-diaminobutane (BDA) and sebacic acid (SA) are massively-produced monomers used for the renewable production of a variety of important PAs that can be obtained by metabolically engineered microorganisms from simple sugars [28,29]. Microbial bio-based monomers produced from simple sugars would provide economic and sustainability benefits [30].

**Scheme 1:**
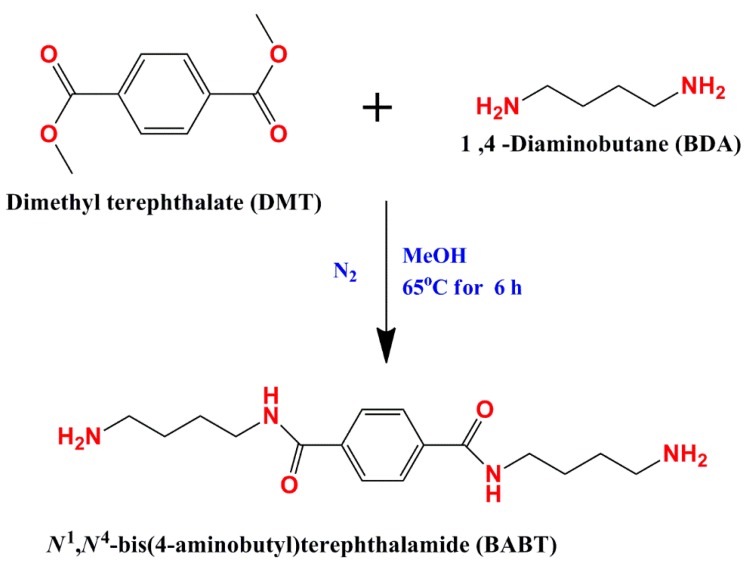
Synthesis of *N*^1^,*N*^4^-bis (4-aminobutyl) terephthalamide (BABT) monomer.

**Scheme 2:**
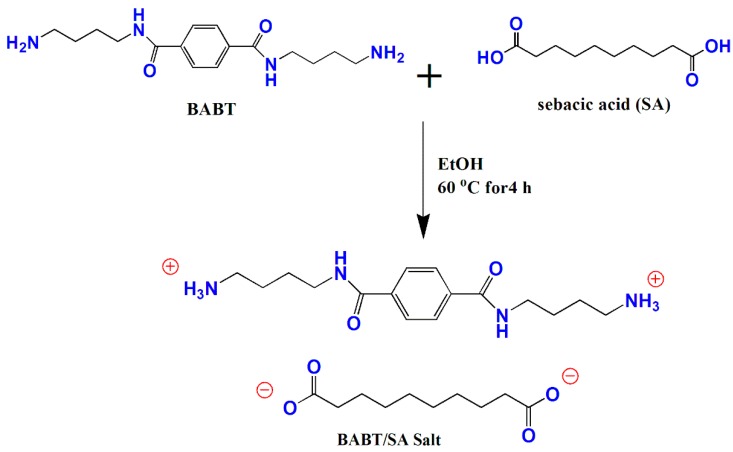
Synthesis of polyamide salt, a BABT/sebacic acid (SA) salt.

Given this interest, the novel bio-based *N*^1^,*N*^4^-bis(4-aminobutyl) terephthalamide (BABT) diamine monomer and BABT/SA polyamide salt can be prepared from the above bio-based monomers: DMT, BDA, and SA, as shown in Scheme 1 and Scheme 2. Gaymans and Fukushima previously reported the synthesis of BABT diamine monomer [31,32]. However, no literature has reported the synthesis of long-chain aromatic polyamide salt (BABT/SA) from BABT diamine and nor are there reports about the synthesis of polyamides from BABT/SA monomer. In the present investigation, we report the synthesis of BABT diamine and BABT/SA salt from bio-based monomers, where the novel long-chain BABT/SA salt was used as the comonomer for the preparation of a series of PA6 copolyamides.

The goal of this study was to determine the effects of the thermal and mechanical properties of PA6 by varying the weight content of the novel long-chain aromatic BABT/SA salt. The PA6-BABT/SA aliphatic-aromatic copolyamides were synthesized by melt condensation of mixed CPL/BABT/SA. The BABT diamine and BABT/SA, as well as the resulting copolymers based on BABT/SA, were characterized using Fourier transform infrared (FT-IR) spectroscopy, hydrogen nuclear magnetic resonance (^1^H NMR), carbon NMR (^13^C NMR), Differential Scanning Calorimetry, X-ray diffraction, thermogravimetric analysis, dynamic mechanical analysis, and mechanical analysis. The novel bio-based aliphatic-aromatic copolyamides exhibited low *T*_m_ (melting temperature) and *T*_g_ (glass transition temperature) values around human body temperature, good thermal stability similar to PA6, and more specifically, increased tensile strength and elongation at break, making them suitable for common engineering applications, hot melt adhesive, and shape memory applications.

## 2. Experimental Details

### 2.1. Chemicals

The following chemicals were purchased from Sigma Aldrich (St. Louis, MO, USA): dimethyl terephthalate (DMT, 99%), 1,4-diaminobutane (BDA, 99%), sebacic acid (SA, 99%), ethanol (EtOH, ≥99.5%), methanol (MeOH, ≥99.8%), diethyl ether (≥99%), toluene (≥99.8%), deuterium oxide (D_2_O, ≥99.9%), sulfuric acid (≥99.99%), formic acid (FA, ≥95–97%), and trifluoroacetic acid-d (TFAA, ≥99.5%). ε-Caprolactam (CPL, 99%) was purchased from Alfa Aesar (Tewksbury, MA, USA) and 6-aminocaproic acid was obtained from TCI (Tokyo Chemical Industry Co., Ltd, Tokyo, Japan).

### 2.2. Methods

#### 2.2.1. Synthesis of *N*^1^,*N*^4^-bis(4-aminobutyl) Terephthalamide Monomer (BABT)

The BABT monomer was synthesized according to the procedure described in the literature [30] from the reaction of DMT with BDA and is illustrated in Scheme 1. In a typical experiment, 22.3 g of DMT was first solubilized in 100 mL of methanol in a three-necked round bottom flask equipped with a magnetic stirrer, nitrogen inlet, and outlet. After the mixture was completely dissolved, 120 mL of BDA was fed into the flask. Subsequently, the reaction mixture was magnetically stirred at 65 °C for 6 h under the persistent flow of nitrogen and kept at room temperature overnight. Afterward, the reaction mixture was filtered. The filtrate was concentrated with vacuum distillation. After evaporation of the solvent, BABT appeared as a white solid, which was dissolved in ethanol and then poured into 300 mL of diethyl ether. The white BABT solid was filtered, soaked in 300 mL of toluene, and dried under vacuum at 80 °C for 12 h, with a yield of 78.1%.

^1^H NMR (D_2_O-d containing 0.25% H_2_SO_4_, 400 MHZ) δ (ppm): 7.5 (s, 4H, Ar), 3.20 (t, 4H, NH–**CH_2_**), 2.74 (t, 4h, **CH_2_**–NH_2_), 1.55–1.35 (m, 8H, **CH_2_** in BDA unit). FT-IR (KBr, cm^−1^): 3342 and 3299 (V_N–H_ stretching of primary and secondary amide) [33], 2957 and 2870 (CH asymmetric and symmetric stretching vibration), 1623 (Amide-I, V_C=O_), 1543 (Amide-II, V_C–N_).

#### 2.2.2. Synthesis of BABT-SA Polyamide Salt

The polyamide salt of BABT-SA was synthesized from the reaction of BABT with SA, as illustrated in Scheme 2. To a solution of BABT (25.7 g, 83.4 mmol) in ethanol (200 mL) at 60 °C, a solution mixture of SA (16.88 g, 83.4 mmol) in ethanol (200 mL) was added dropwise. After completion of the addition, the resulting mixture became clear and the reaction mixture was stirred at 60 °C for another 4 h. Then, the solution was stirred at 30 °C overnight and the formed precipitate was filtered and finally dried at 80 °C in vacuum for 12 h to yield white powders, viz. BABT/SA (yield: 96%).

^1^H NMR (D_2_O-d, 400 MHZ) δ (ppm): 7.5 (s, 4H, Ar), 3.32 (t, 4H, **CH_2_**–NH–CO–), 2.95 (t, 4H, **CH_2_**–NH_3_^+^), 2.01 (t, 4H, **CH_2_**–COO^−^), 1.65 (t, 8H, NH–CH_2_–**CH_2_–CH_2_**–CH_2_–NH_3_^+^), 1.35 (t, 4H, CH_2_–**CH_2_**–COO^−^), 1.13 (s, 8H, CH_2_ in SA unit). FT-IR (KBr, cm^−1^): 3323 and 2126 (V_NH3+_, V_N-H_), 1550–1630 (overlapping of carboxylate and ammonium ion), 1746 and 1120 (transverse rotation), 1411 (V_COO_^−^).

#### 2.2.3. Synthesis of PA6-BABT/SA Copolyamides

**Scheme 3:**
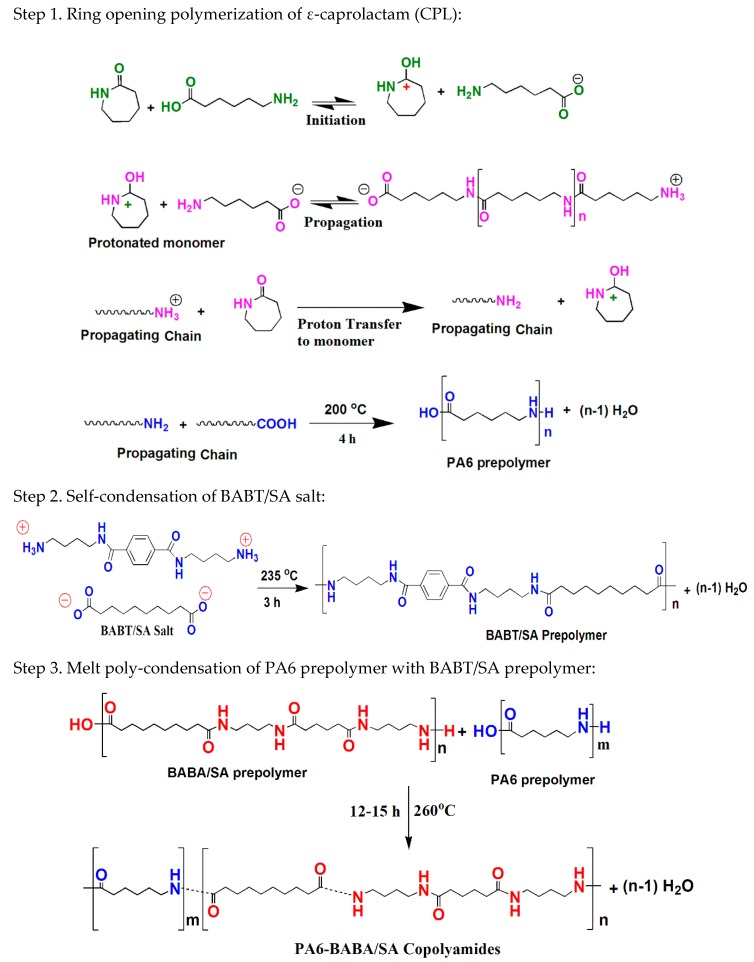
Steps 1–3 of the synthesis of polyamide 6 (PA6)-BABT/SA copolyamide based on ε-caprolactam with long chain aromatic BABT/SA salt.

**Scheme 4:**
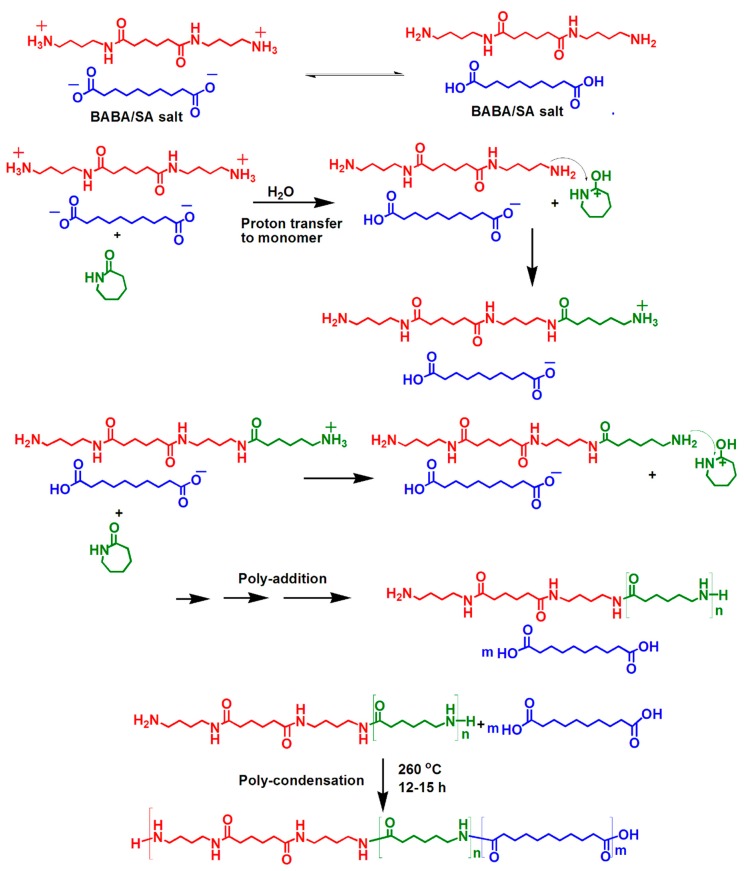
Poly-addition initiated by amino group at one side of BABT and the subsequent poly-condensation with SA.

**Scheme 5:**
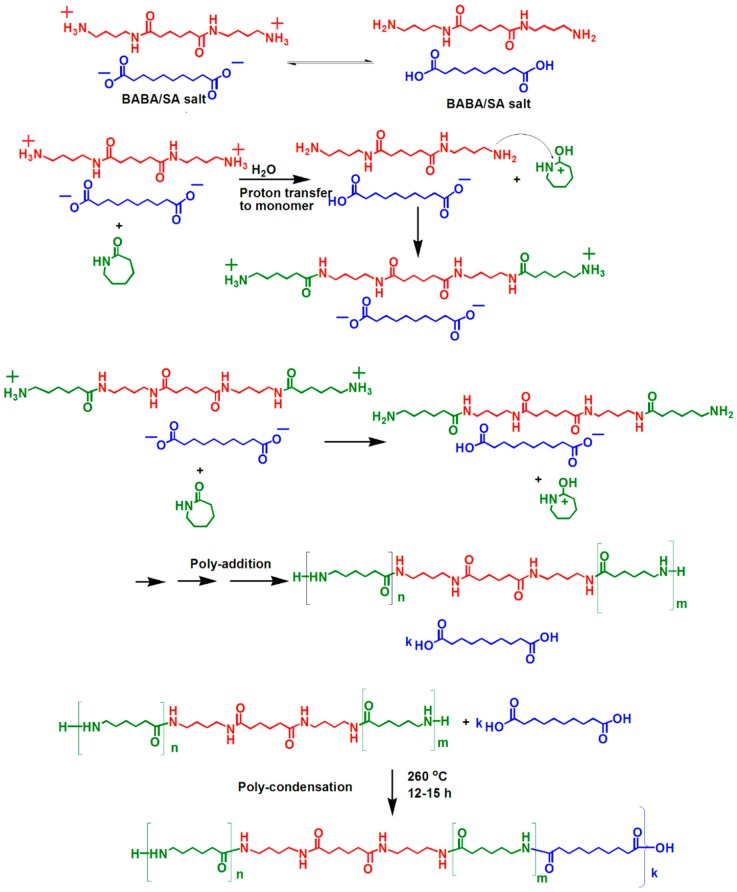
Poly-addition initiated by amino group at both sides of BABT and following the poly-condensation with SA.

The structure and possible synthetic procedure of the PA6-BABT/SA copolymers with ε-caprolactam (CPL) and BABT/SA salt are depicted in Scheme 3, Scheme 4 and Scheme 5. The polymerization was performed in a Dean-Stark type flask equipped with a central mechanical stirrer, a nitrogen inlet and outlet, a distillation head connected to a condenser, and a receiver flask. For the synthesis of PA6-BABT/SA copolyamides, the as-synthesized BABT/SA salt (10, 20, and 30 wt %) was mixed with CPL (90, 80, and 70 wt %) and distilled water (20 wt %). The initiator 6-aminocaproic acid (ACS, 0.1 wt %) was also added to the flask for the polymerization reaction. The reaction flask was placed in an oil bath at 235 °C for 3 h and the temperature was raised to 260 °C and kept constant for 12 h under a persistent nitrogen flow. After the reaction, the polymer was ground into a powder and washed with distilled water at 80 °C for 12 h. The polymer was dried in a vacuum oven at 80 °C overnight. The yield was 94.2%.

^1^H NMR (TFAA, 400 MHZ) δ (ppm): 7.5 (s, 4H, Ar), 3.61-3.50 (d, 10H, **CH_2_**–NH–CO–), 2.82–2.54 (d, 6H, NH–CO–**CH_2_**), 1.7–1.13 (m, 26H, **CH_2_**). FT-IR (KBr, cm^−1^): 3302 (V_N-H_), 3065 (V_N-H_and V_C-N_), 2952–2857 (CH asymmetric and symmetric stretching vibration), 1630 (Amide-I, V_C=O_), 1536 (Amide-II, V_C–N_ stretch and V_N–H_ bend), 1460 (Amide-III, V_C–N_, δ_C–H_), 1255 (Amide-IV, V_C–C=O_), 686 (Amide-V, V_N–H_, out-of-plane bending).

### 2.3. Measurements

#### 2.3.1. Nuclear Magnetic Resonance Spectroscopic Analysis

The room temperature ^1^H NMR spectrum of BABT, BABT/SA, and PA6-BABT/SA copolyamides were recorded in D_2_O and TFAA using a Bruker Avance 400 MHZ spectrometer (Billerica, MA, USA). The solvent signal was used as a reference.

^13^C NMR spectra were recorded using a Bruker Avance 400 MHZ spectrometer. Samples (10 wt %) were dissolved in TFAA solvent in 10 mm tubes. Routine acquisitions at 25 °C involved a 1.3 s acquisition time, a 45° pulse width of 5.8 μs, and a 5 s recycle delay. The number of accumulated transients was 12,800, requiring a collection time of 10 to 13 h. Raw data were zero-filled up to 12,800 points and filtered using 1 Hz line broadening. Quantitative analysis of the peak integrals was completed after baseline correction and deconvolution of the overlapping peaks.

#### 2.3.2. Fourier Transform Infrared Spectroscopic Analysis

The FT-IR spectra of samples were investigated using a Nicolet 5700 FT-IR spectrometer (Waltham, MA, USA). All samples were measured with an average signal of 32 co-added scans using a resolution of 4 cm^−1^, in the wave number range of 500–4000 cm^−1^ using a KBr pellet.

#### 2.3.3. Viscometry

Intrinsic viscosity measurements were performed with 0.5 g/dL polymers in concentrated sulfuric acid (H_2_SO_4_, 96%) at 25 ± 0.05 °C using an Ubbelohde viscometer (Koehler instrument company, Holtsville, NY, USA). The value of flow time was an average of three values with a standard deviation within ±0.2 s. The flow time of H_2_SO_4_ and each of the polymer solutions were used to calculate the relative and specific viscosities. Single point intrinsic viscosities were then calculated using the Solomon and Ciuta relationship:

(1) $$\;\left[\eta \right]=[2\left(\eta_{sp}-ln\eta_{rel}{)}^{1/2}\right]/C\;$$

where $\eta_{sp}$ is specific viscosity, $\eta_{rel}$ is relative viscosity, and C is the sample concentration [34].

#### 2.3.4. Gel Permeation Chromatography (GPC)

The number-average molecular weight (*M*_n_), weight-average molecular weight (*M*_w_), and polydispersity index (PDI) of the polymer samples (10 mg/5 mL) were determined via the gel permeation chromatography (GPC) device from a Waters Alliance GPC/V2000 system (Malvern Ltd, Malvern, UK) equipped with refractive index (RI) and water high-resolution (HR) column at a flow rate of 1.0 mL/min and injection volume of 100 μL. Column temperature was held at 30 °C. The well-characterized narrow poly (methyl methacrylate) (PMMA) in hexafluoroisopropanol (HFIP) was used as the calibration standard.

#### 2.3.5. Differential Scanning Calorimetry (DSC)

A Differential Scanning Calorimetry (SHIMADZU AGS-X 500 N, Waltham, MA, USA) was used to analyze the melting temperature of the monomer and polymer samples. Samples of 3–5 mg were encapsulated onto the aluminum crucibles. The samples were first heated from 60 to 250 °C, maintained for 3 min, then cooled to 60 °C. All the samples were subjected to an underlying heating rate of 10 °C/min in all cases.

#### 2.3.6. Thermogravimetric Analysis (TGA)

Thermogravimetric analysis was performed with 5–10 mg samples, at a heating rate of 10 °C/min under a nitrogen atmosphere using a TGA Q500 (TA Instrument, Newcastle, DE, USA).

#### 2.3.7. X-ray Diffraction Analysis

The powder X-ray diffraction pattern was registered using a Bruker D8 Discover powder diffractometer (Cu Kα radiation, λ = 0.1542 nm, voltage 40 KV, 100 mA, Malvern Ltd, Malvern, UK). The testing specimen samples were scanned from 10° to 50° of 2θ with a scanning speed range of 0.2°/min.

#### 2.3.8. Dynamic Mechanical Analysis (DMA)

Dynamic Mechanical Analysis (DMA) was used to analyze the thermomechanical properties of the copolyamides (TA Instruments, Q 800, Newcastle, DE, USA). Temperature dependence of the loss factor tan δ was measured by heating a sample (size: 15 mm × 15 mm × 2 mm) from –20 °C to 150 °C at a heating rate of 10 °C/min and a frequency of 3 Hz.

#### 2.3.9. Tensile Test

The static mechanical properties of the copolyamides were determined at 25 °C according to ASTM D638 (Dumbbell shaped, 25 mm × 6 mm × 2 mm) using an INSTRON 3800R electronic tensile tester (Norwood, MA, USA). The tests were conducted at a crosshead speed of 50 mm/min. At least five specimens of each polymer were analyzed for an average value.

#### 2.3.10. Ultraviolet (UV)-Visible Spectrophotometer

The films were obtained at 200 °C at a hydraulic pressure of 10 MPa and the thickness of the film was 30 μm. The transmittance of the polymers films was measured using an ultraviolet (UV)-visible spectrophotometer (Waltham, MA, USA). The UV absorption was measured using an ultraviolet spectrophotometer with the range of 180–400 nm.

## 3. Results and Discussion

### 3.1. Synthesis and Structural Characterization of the Monomers

**Figure 1:**
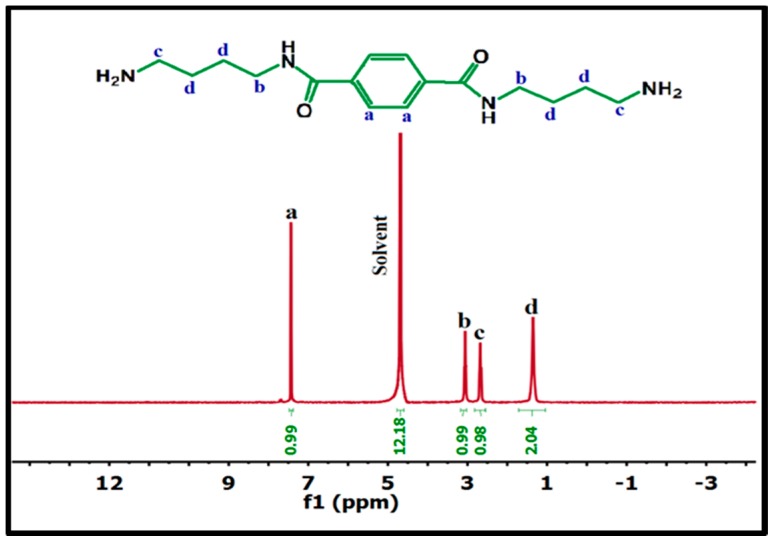
Hydrogen nuclear magnetic resonance (^1^H NMR) spectra of *N*^1^,*N*^4^-bis(4-aminobutyl) terephthalamide diamine (BABT) diamine.

**Figure 2:**
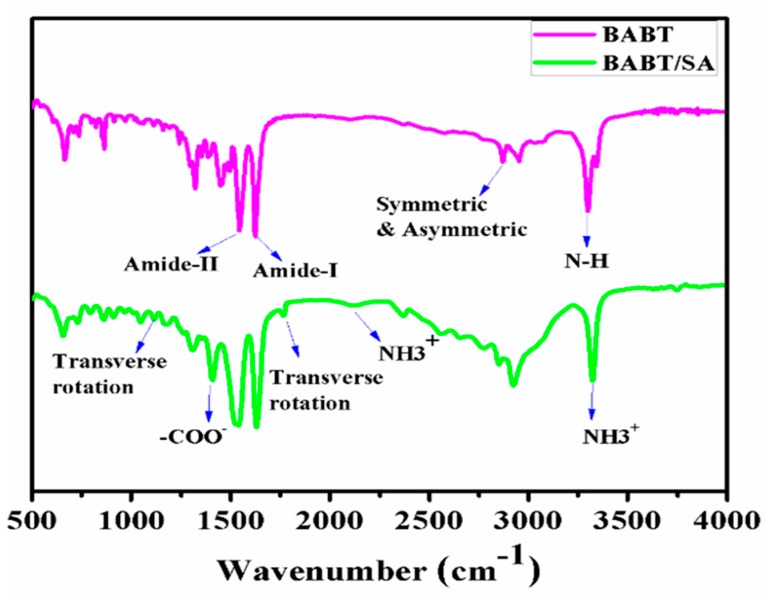
Fourier transform infrared (FT-IR) spectra of BABT diamine and BABT/sebacic acid (SA) salt.

**Figure 3:**
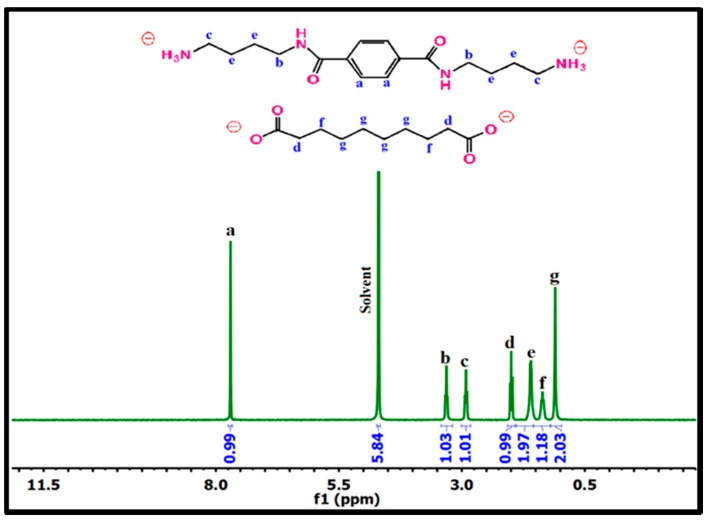
^1^H NMR spectra of BABT/SA salt.

A BABT diamine was synthesized based on a procedure described in the literature, as shown in Scheme 1. The ^1^H NMR spectrum of BABT (Figure 1) shows that the resonance at 7.5 ppm is attributed to the characteristic protons of the aromatic moiety (4H, s, Ar). The signals at 3.20 ppm (t, 4H, NH–**CH_2_**) and 2.74 ppm (t, 4H, **CH_2_**–NH_2_) indicate the formation of amide groups in the aromatic monomer. These results confirm the occurrence of the reaction between amine (present in BDA) and alkyl units (present in DMT). Figure 2 (pink curve) depicts the FT-IR spectra of BABT diamine showing good agreement with its chemical structure. In addition, the polyamide organic salt of BABT/SA, composed of BABT and SA with stoichiometric composition, was obtained simply by mixing the two components with the molar ratio of 1:1, which is illustrated in Scheme 2. The salt formation between the BABT and SA was confirmed by FT-IR. The representative FT-IR spectrogram is displayed in Figure 2 (green curves) and ^1^H NMR spectroscopy representative spectrogram is displayed in Figure 3.

### 3.2. Synthesis and Structure Analysis of Aliphatic-Aromatic Copolyamides

For the first time, new aliphatic-aromatic copolyamides were synthesized via melt polymerization of different wt % of long-chain aromatic BABT/SA salts with CPL, as described in detail in the above experimental section (Scheme 3, Scheme 4 and Scheme 5). Three expected mechanisms for the preparation of PA6-BABT/SA random copolymides are proposed and shown in Scheme 3, Scheme 4 and Scheme 5. The prepolymerization was first allowed to proceed at 235 °C for three hours. In Scheme 3, three step reactions are required for the PA6 copolymerization. In Step 1, 6-aminocaproic acid (ACS) acts as an activator to initiate the polymerization reaction. In this typical reaction (Step 1), the free proton of COOH present in the ACS first transfers to the carbonyl of CPL to form an active center. Then, the amine nitrogen or amine end group of the growing chain of ACS attack the carbonium ion of CPL through a nucleophilic addition reaction and rapidly initiate the following poly-addition. In Step 2, the NH_3_^+^ (quaternary ammonium) group in the BABT/SA salt reacts with a COO^−^ group through a self-condensation reaction and initiate the prepolymerization. In Step 3, by increasing the temperature to 260 °C, the chain is terminated by the poly-condensation of amine and carboxylic end groups. 

As shown in Scheme 4, the BABT/SA salt acted as a catalyst and initiated the copolymerization reaction. In this typical reaction, in the presence of BABT/SA salt, the free proton of NH_3_^+^ in the BABT/SA salt first transfers to the carbonyl of ε-caprolactam and rapidly initiates the poly-addition. The two amino groups of BABT more likely initiate the nucleophilic addition to extend the molecular chain on both sides (Scheme 5). Following that, poly-condensation occurs between the linear molecules and SA unit to further increase the molecular weight of the polymer. SA might be randomly arranged in the copolyamides after amide exchange [35,36]. Scheme 3, Scheme 4 and Scheme 5 outline the most likely synthetic paths based on the polymerization chemistry. 

**Figure 4:**
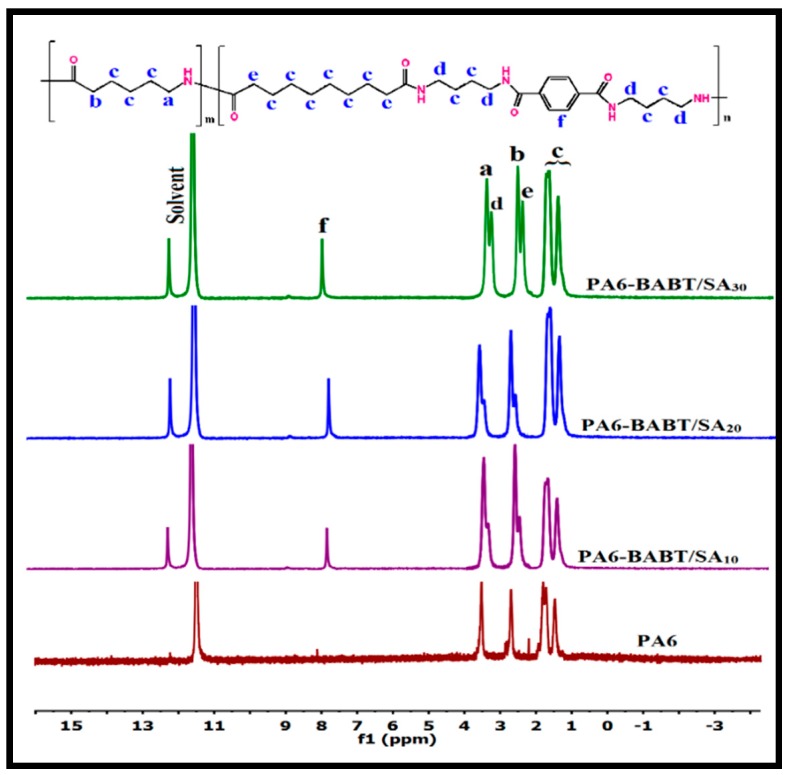
^1^H NMR spectra of PA6 and PA6-BABT/SA copolyamides.

The chemical structure of all polyamides were confirmed by ^1^H NMR, FT-IR, and ^13^C NMR spectroscopy. Figure 4 shows the representative ^1^H NMR spectra of neat PA 6. The peaks in the region of 3.61 ppm and 2.82 ppm were ascribed to the chemical shifts of the methyl protons adjacent to the amino group (**CH_2_**–NH–CO–) and the carbonyl group (NH–CO–**CH_2_**) of PA6, respectively. The proton signals in the 2.08–1.30 ppm region are attributed to the other protons of the aliphatic methylene units of PA6. After the reaction of PA6 with BABT/SA, the new peaks appeared in the region of 7.78, 3.50, and 2.54 ppm, indicating the successful incorporation of BABT/SA salt into the PA6. The adding ratio of BABT/SA salt varied from 10 to 30 wt %, and the peak intensity of the region at 7.78 ppm and 2.54 ppm increased correspondingly. Based on the above discussions, the PA6-based copolymer was successfully synthesized, in which the BABT/SA segments were randomly arranged. 

**Figure 5:**
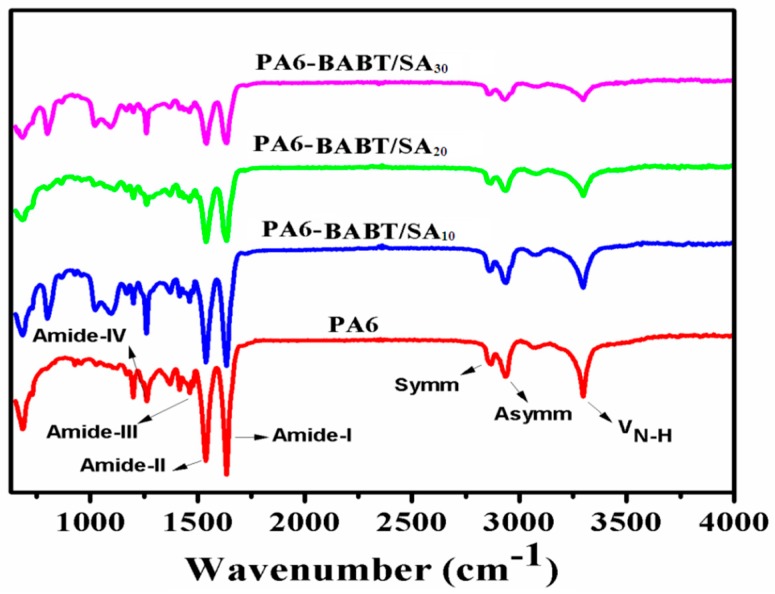
FT-IR spectra of PA 6 and PA6-BABT/SA copolyamides.

The FT-IR spectra of PA6 and PA6-BABT/SA copolymers are shown in Figure 5. PA6-BABT/SA copolyamides exhibited almost identical infrared characteristics as PA6. The absorption band at 3302 cm^−1^ is ascribed to the hydrogen bonded V_N–H_ stretching vibration. The coupled absorption band at 2857 (symmetric) and 2952 cm^−1^ (asymmetric) are attributed to the stretching vibration of V_C–H_. The signals at 1630, 1536, 1460, 1255, and 686 cm^−1^ are attributed to amide-I (V_C=O_), amide-II (V_C__–N_ stretch and V_N__–H_ band), amide-III (V_C–N_ and δ_C–H_), amide-IV (V_C–C=O_), and amide-V (V_N–H_, out-of-plane bonding vibration), respectively [37]. Nonetheless, for the copolyamides with an increasing weight ratio of BABT/SA, these amide absorption bands appeared weaker, which denotes a reduction in the crystallinity order. The FT-IR and ^1^H NMR results suggest that the desirable PA6-BABT/SA copolyamides were successfully synthesized. 

**Figure 6:**
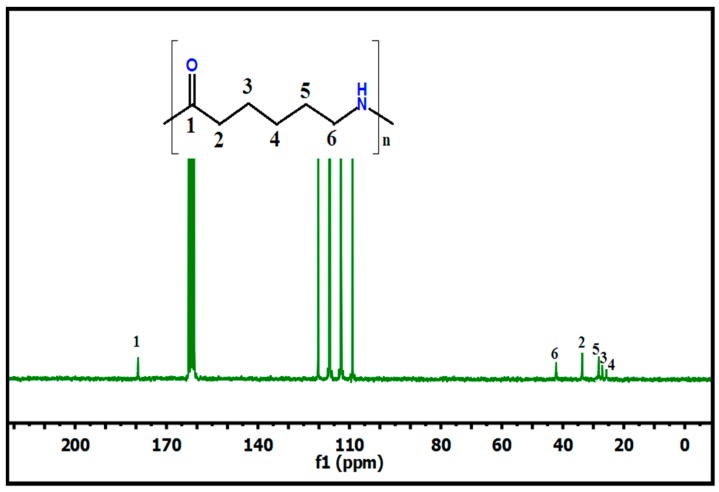
^13^C NMR spectrum of neat PA6 recorded in trifluoroacetic acid-d (TFAA) solvent.

**Figure 7:**
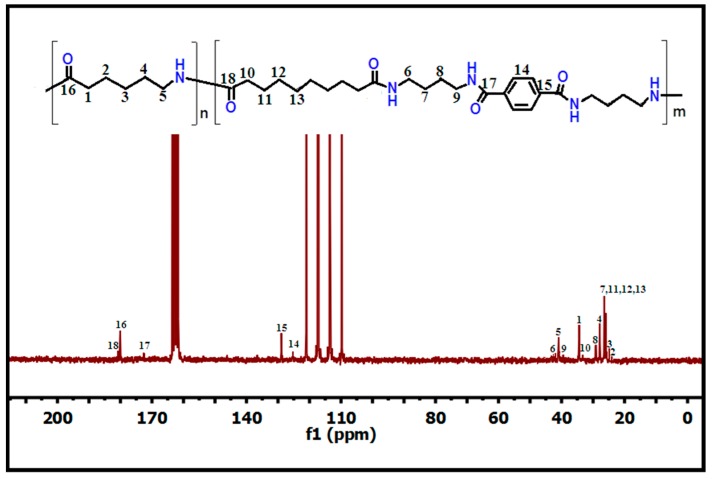
^13^C NMR spectrum of PA6-BABT/SA_30_ copolyamide recorded in TFAA solvent.

In order to obtain more detailed information about the structure of PA6-BABT/SA copolyamides, ^13^C NMR was performed. ^13^C NMR spectra were also in good agreement with the expected structures, as shown in Figure 6 and Figure 7. The chemical shift and assignments are summarized in Table 1.

**Figure 8:**
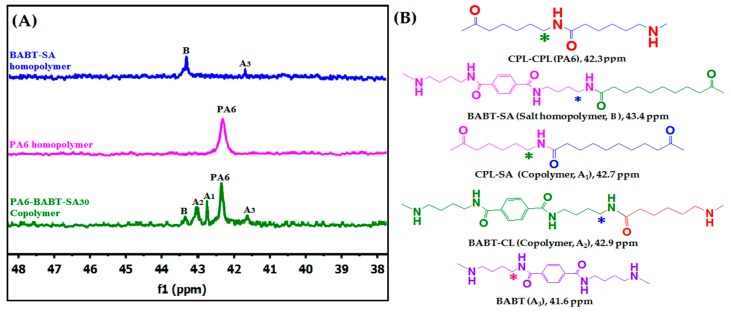
(**A**) ^13^C NMR Expanded spectra of BABT-SA, PA6, and PA6-BABT/SA_30_ recorded in TFAA solvent. (**B**) Assignment of the carbonyl resonances (marked with an asterisk) in the PA6-BABT/SA copolyamides for possible dyad sequences.

Figure 8A displays the ^13^C NMR spectra of BABT/SA homopolymer, neat PA6, and PA6-BABA_30_. Based on the carbon resonances of BABT-SA and PA6, the five methylene carbon peaks located at 41.6, 42.3, 42.7, 42.9, and 43.4 ppm are present in the spectra of the copolyamides (Figure 8), which correspond to the five possible dyad arrangements of the PA6 and BABT/SA units (BABT, CPL-CPL, CPL-SA, BABT-CPL, and BABT-SA) along the polymer chain. The chemical structures and peak areas of CPL-CPL, BABT, BABT-SA, BABT-CPL, and CPL-SA dyad sequences in the PA6-BABT/SA copolyamides are shown in Figure 8B. These results enabled the calculation of the degree of randomness (R) of PA6-BABT/SA copolyamides using Equations (2)–(5) [23]:

(2) $$\;F_{Copolyamides}=\;A_{1}+A_{2}+A_{3}\;$$

(3) $$\;F_{CPL}=\;A_{1}+PA6\;$$

(4) $$\;F_{BABT/.SA}=\;A_{2}+A_{3}+B\;$$

(5) $$\;R=\;F_{Copolyamides,\;\;total}/\;\left(2\cdot F_{CPL}\cdot F_{BABT/SA}\right)\;$$

Then, Equation (6) was used to calculate the molar percentage of BABT/SA salt present in the PA6-BABT/SA copolymers:

(6) $$\;mol\;\%\;BABT/SA=\;F_{BABT/SA}/\;\left(F_{CPL}+F_{BABT/SA}\right)\;$$

where $F_{Copolyamides}$, $F_{CPL}$, and $F_{BABT/SA}$ represent the molar fractions of the total amounts of PA6-BABT/SA copolyamides, CPL, and BABT/SA salt, respectively. $A_{1}$, $A_{2\;},\;\mathrm{and}\;A_{3}$ PA6 and B represent the peak areas of the corresponding shifts. R represents the degree of randomness. The corresponding calculated results are listed in Table 2.

An R value greater than one indicates that the units have a more alternating tendency. When R is less than one, the units tend to cluster in homogenous sequences and thus the copolymer exhibits a block character. When R is two, then a completely alternating copolymer is present. In the case of R being zero, the copolymer is a pure block copolymer, or the system is a mixture of two polyamides. As presented in the last column of Table 2, the total degree of randomness (R) for PA6-BABT/SA_20_ and PA6-BABT/SA_30_ was calculated as 1.05 and 1.03, respectively, which reveals that the random copolyamides were prepared. 

It was not possible to calculate the composition of PA6-BABT/SA_10_ copolyamide from ^13^C NMR due to the very low intensities of the BABT/SA salt peaks. 

### 3.3. Intrinsic Viscosity and Molecular Weight

**Figure 9:**
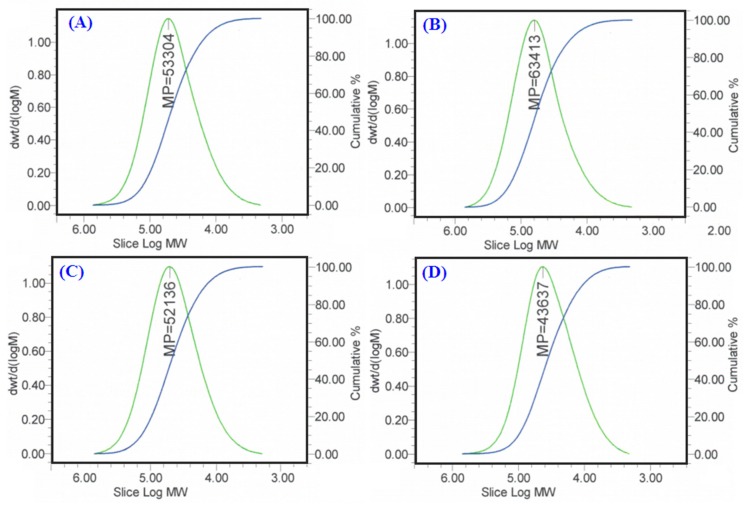
Gel permeation chromatography (GPC) chromatographs of (**A**) neat PA6, (**B**) PA6-BABT/SA_10_, (**C**) PA6-BABT/SA_20_, and (**D**) PA6-BABT/SA_30_.

The viscosity method was used to measure the single point intrinsic viscosities of PA6-BABT/SA copolyamides in concentrated sulfuric acid (96%) using an Ubbelohde viscometer. *M*_n_, *M*_w_, *M*_p_, and PDI (polydispersity index) were measured using GPC. *M*_p_ is the molecular weight corresponding to that of the maximum of the chromatographic peak. Additionally, Mark-Houwink constants *K* and *α*, in Equation (7), were obtained from log-log plot of single point intrinsic viscosity versus *M*_w_ (GPC measured):

(7) $$\;\left[\eta \right]=KM_{\eta }^{\alpha }\;$$

where *K* and α are constants associated with the interaction between the polymer and the solvent. Mark-Houwink constants were obtained from the slope and intercept of the plot as *K* = 2.169 × 10^−4^ and α = 0.778 for sulfuric acid (96%). An α value greater than 0.75 is typical for good solvents. Table 3 shows the results obtained from the PA6-BABT/SA copolyamides, whereas Figure 9 shows the chromatographs for the neat PA6 and PA6-BABT/SA copolyamides. 

Intrinsic viscosities and molecular weights of the copolyamides increased with the increasing incorporation of BABT/SA, reaching a maximum value when BABT/SA content was 10 wt %. The value then began to decrease when BABT/SA was above 20 to 30 wt %. Because the polymerization rate of the aromatic BABT/SA salt was faster than that of the neat PA6, the copolymerization rate thus increased with the incorporation of 10 wt % aromatic BABT/SA salt, which resulted in a higher molecular weight copolymer, reflected by the greater intrinsic viscosity. However, the copolymerization may occur in the presence of an excessive amount of BABT/SA salt, inhibiting the block-polymerization process and reducing the molecular mass of the copolyamides. On the other hand, polyamide salts from BABT and SA have a catalytic influence on the polymerization of CPL. Thus, the polymerization reaction with an increasing amount of BABT/SA was much faster, and the molecular weight and intrinsic viscosity were lower than those of the neat PA6. Moreover, by varying the weight content of BABT/SA in the reactants, the intrinsic viscosity of the final products ranged from 1.33 to 0.94 dL/g and the *M*_n_ as well as *M*_w_ varied from 30,900 to 23,600 Da and 77,800 to 48,700 Da, respectively. The range of PDI for the PA6-BABT/SA copolyamides was between 2.11 and 2.06. Generally, PA6 with *M*_n_ higher than 13,000, endows good physical and mechanical properties, which specifically befits applications in plastic, membrane, and textile yarn, and so on [38].

### 3.4. Thermal Properties

**Figure 10:**
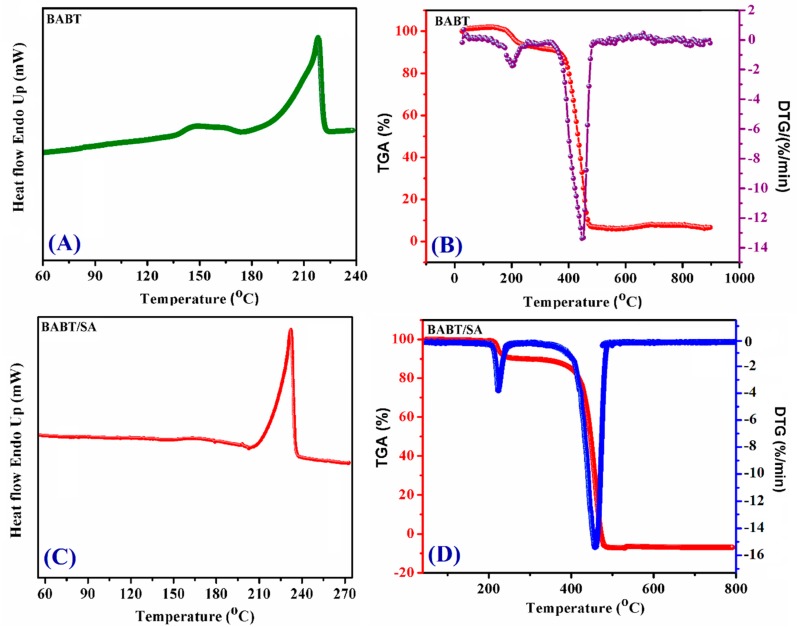
Differential scanning calorimetry (DSC) traces of (**A**) BABT and (**C**) BABT/SA salt; thermogravimetric analysis (TGA) of (**B**) BABT and (**D**) BABT/SA salt.

DSC and TGA techniques were applied to investigate the melting temperature and thermal stability of BABT, BABT/SA, and polyamides, and the results are summarized in Table 4. The polymerization process strongly depends on the thermal properties of the synthesized monomers. Figure 10A shows that the *T*_m_ of BABT is 217.1 °C. From the TGA thermogram (Figure 10B), BABT shows a decomposition temperature (*T*_d_) at 5 wt % loss (*T*_5%_) at 194.25 °C (elimination of water) and 56 wt % loss at 431 °C. DTG (differential thermogravimetric) curves of BABT (Figure 10B) showed two main peaks with maximum degradation temperatures (*T*_max_) of 203.3 and 447.7 °C. Figure 10C,D demonstrate that the *T*_m_ of BABT/SA is 231.4 °C and the *T*_5%_ (5 wt % loss) is 221.8 °C (elimination of water). BABT/SA presents a *T*_max_ at 223.1 and 457.6 °C (Figure 10D).

**Figure 11:**
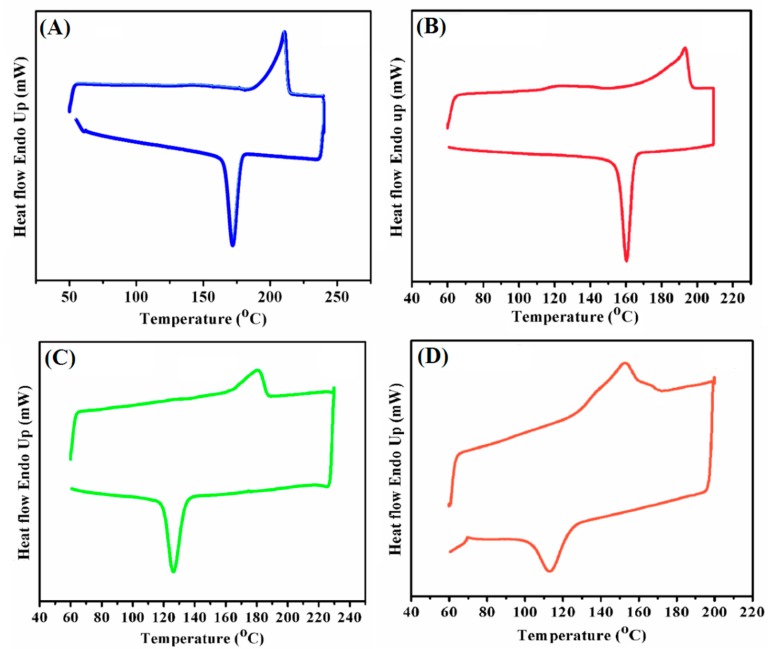
DSC analysis of (**A**) PA6, (**B**) PA6-PABT/SA10, (**C**) PA6-BABT/SA20, and (**D**) PA6-BABT/SA30 copolyamides.

The thermal and melt crystallization properties of the polymers directly influence their usage and processing performance, which were also analyzed by DSC. The DSC traces are shown in Figure 11 and the data extracted from these traces are summarized in Table 4. The *T*_m_ of the copolymer decreased from 210.7 to 152.5 °C (Figure 11A–D) when the addition of BABT/SA salt increased from 10 to 30 wt %. Similarly, the observed melting enthalpies (Δ*H*_m_, and hence the crystallinity X_c%_) were lower (46.9 to 38.6 J/g) compared to that of neat PA6 (51.1 J/g). Furthermore, the melting endotherms of copolyamides containing 20 to 30 wt % BABT/SA were broad in contrast to the reference PA6. The broadening of the melting endotherms is related to a larger distribution of crystallinity and crystal perfection. These results indicate that neat PA6 is not co-crystalline with the BABT/SA unit. The reduction in the *T*_m_ of the copolyamides may be ascribed to the existence of an aromatic moiety in the polymer main chain, which interrupts the intermolecular hydrogen bonding and destroys the molecular chain regularity, therefore resulting in a crystallization reduction. This indicates that BABT/SA series up to 30 wt % act as impurities, which inhibit the construction of PA6 crystals. The lower *T*_m_ may contribute to the mobility of copolyamides and improve its processability. 

We noticed that the DSC curves of the copolymers with 10 to 30 wt % BABA/SA salt displayed a narrow crystallization peak from 171.6 to 113.4 °C, as shown in Figure 11A–D. Beyond that, the DSC traces in Table 4 show that the crystallinity of all PA6-BABT/SA copolyamides is lower than that of neat PA6, and decreases with escalating BABT/SA weight content. As such, good transparency of these copolymers is highly expected. The large steric hindrance of aromatic radicals and the copolymerization disrupting the well-defined structures and hydrogen bonding may be responsible for this observation. In addition, the *T*_m_ of the commercially available PA11 and PA12 are about 190 and 180 °C [39], respectively, which is almost the same as the PA6-BABT/SA_10_ and PA6-BABT/SA_20_ copolyamides.

**Figure 12:**
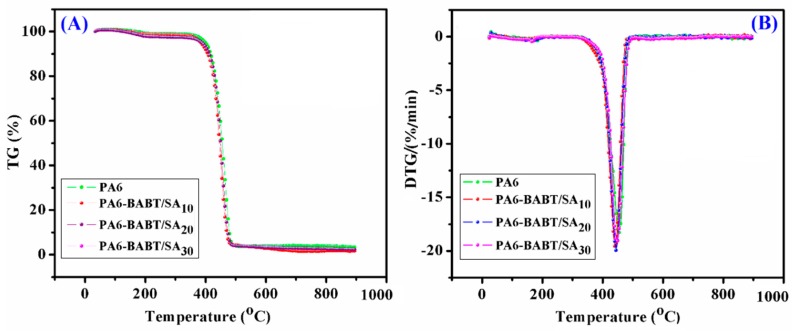
(**A**) TGA of PA6 and PA6-BABT/SA copolyamides. (**B**) DTG (differential thermogravimetric) curves of PA6 and PA6-BABT/SA copolyamides.

The thermal stability of the synthesized copolyamides was assessed using TGA analysis. The temperature of 5% weight loss (*T*_5%_) and the maximum decomposition temperature (*T*_max_) of these copolyamides are listed in Table 4. No significant transformation was observed in the curves of Figure 12A by varying BABT/SA weight ratios, which indicated that the incorporation of aromatic moieties into the PA6 backbone was noticeably favorable to the thermal stability of the resulting copolymers. The apparent weight loss occurred in the range of 150 to 180 °C due to the water molecules absorbing on the surface of the polymer [40]. The copolyamides were thermally degraded, mainly through a simple step process with a 5% weight loss above 350 °C and a 95% weight loss above 430 °C. DTG curves (Figure 12B) show that the *T*_max_ of the copolyamides was observed in the range of 453.4 to 446.3 °C. The thermal stability of the copolyamides is similar to the commercially available PA6 because of the presence of an aromatic radical in the main chain of the copolymer, which indicates the novel series of copolyamides are suitable for most common applications.

### 3.5. Crystallization Properties

**Figure 13:**
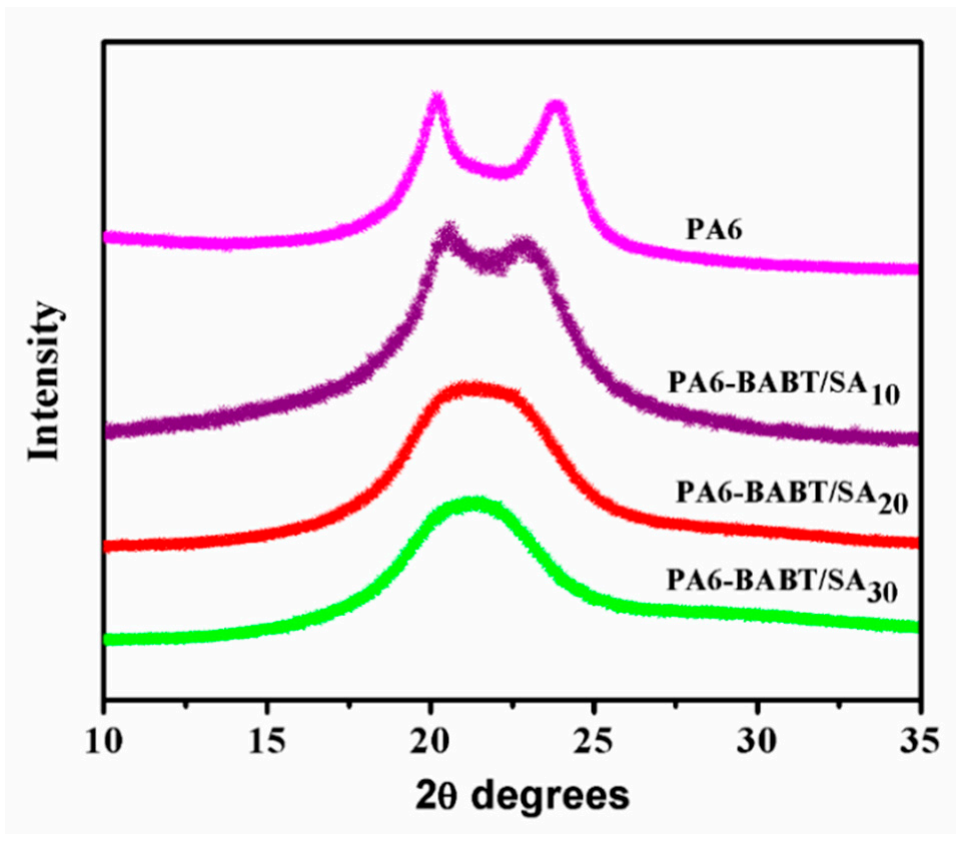
X-ray diffraction (SRD) of PA6 and PA6-BABT/SA copolyamides with different weight contents of BABT/SA salt.

The crystallization behavior of the PA6-BABT/SA copolyamides was further confirmed using XRD. Polyamides have a variety of crystalline structures, of which the α and γ crystalline phases are most common. The α crystalline phase consists predominantly of two characteristic diffraction peaks (reflection at 20.2° and 23.9°), whereas the γ crystalline phase consists predominantly of a single characteristic diffraction peak (shoulder at 21.3°) [41]. As shown in Figure 13, the XRD profile demonstrates that the neat PA6 exhibits the α form (reflection at 20.2° and 23.9°). As the quantity of BABT/SA increased to 10 wt %, two peaks were located at 20.3° and 23.4°, indicating the presence of the α crystalline phase. Further, increasing the BABT/SA content to 20 to 30 wt % did not result in a sharp diffraction peak. The absence of sharp diffraction peaks is due to the low crystallinity of the polymers. The only broad single amorphous peak was observed with a shoulder at 21.2°, which reveals that the copolyamides mainly include the γ crystalline phase. [42]. Transition of the α crystalline phase to the γ crystalline phase also indicates that the crystallizability of the copolyamides weakened with the increase in the aromatic moiety in the polyamide main chain, which is consistent with the DSC traces. These data agree with the FT-IR results.

### 3.6. Thermo-Mechanical Properties of Copolyamides

**Figure 14:**
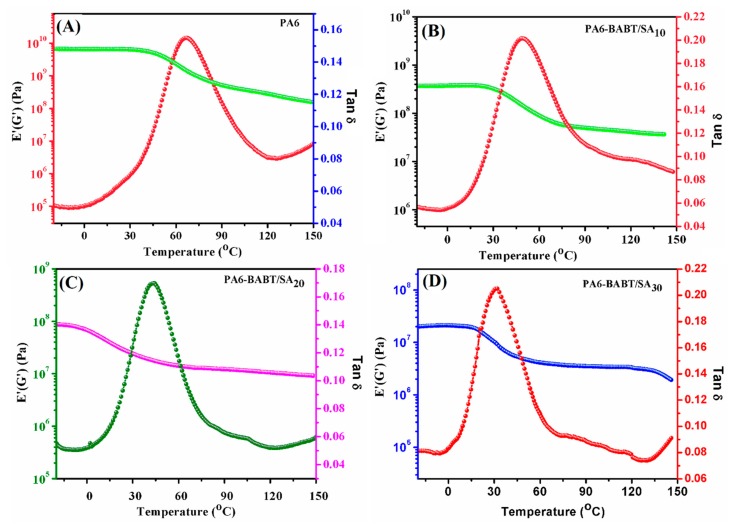
Dynamic mechanical analysis (DMA) curves (Tan δ and storage modulus E’) of (**A**) PA6 and (**B**–**D**) PA6-BABT/SA copolyamides.

**Figure 15:**
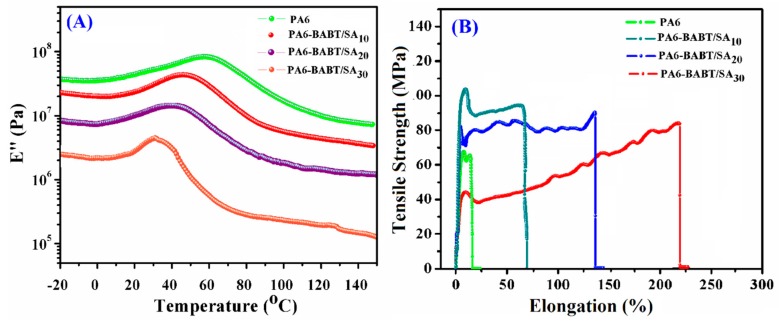
(**A**) DMA curves (loss modulus E”) of PA6 and PA6-BABA/SA copolyamides. (**B**) Tensile strength curves of PA6 and PA6-BABA/SA copolyamides.

DMA is an important and effectual method to study the thermo-mechanical properties of the polymers. The glass transition temperature (*T*_g_), the most salient index to polyamides, is determined from the tan δ maximum. *T*_g_ is intimately related with the composition and comonomer sequence of a copolymer, which is a microscopic expression of internal molecules varying between moving and freezing states [43]. As shown in Figure 14A, the *T*_g_ of PA6 is 68.5 °C, which is higher than long chain aliphatic polyamides. For example, the *T*_g_ of PA 1010, PA 1012, and PA 510 are only in the range of 40 to 50 °C [44]. The molecular structure of PA6 contains more amide bonds with a semicrystalline nature, which limit the appearance of potential molecular chain compatibility to reduce the flexibility of the polymer chain. Therefore, more power is consumed to move the molecular chain from a freezing state, which increases the *T*_g_ value of PA6. When 10 to 30 wt % BABT/SA was introduced into PA6, the reduction in crystallinity decreased the *T*_g_ of PA6-BABT/SA copolyamides. The aromatic region of the BABT/SA units is reflected in the *T*_g_ values, where three copolyamides exhibit different *T*_g_ temperatures, ranging from 49.1 to 31.2 °C (Figure 14B–D). The *T*_g_ of the copolyamides are around human body temperature, which makes a polymer useful for shape memory applications in the medical field [45]. The transition of *T*_g_ to the room temperature range by varying the weight ratio of comonomer (BABT/SA) helps to adjust the working temperature range of the shape memory polymers. Furthermore, the tan δ values are almost the same as obtained by the loss modulus (E”) shown in Figure 15A. The values are listed in Table 5. In addition, the lower storage modulus (E’) of the BABT/SA based copolyamides compared to the linear aliphatic reference polyamide (PA6) indicates that the incorporation of BABT/SA decreases the crystallinity and enhances the flexibility of the copolymers. These results clearly show that the chemistry of BABT/SA salt plays a major role in tuning the thermal properties of the copolyamides.

### 3.7. Mechanical Properties

The typical stress-strain curves for the PA6-BABT/SA and reference PA6 polymers are shown in Figure 15B. The results of the tensile testing are listed in Table 5. PA6 has good tensile strength but the ductility and elasticity are poor. The reason for adding the long-chain aromatic polyamide salt is to improve the tensile strength and ductility of PA6. The mechanical properties of the PA6-BABT/SA copolyamides principally depend on the composition and molecular weight of the copolymers. Therefore, the effect of different weight contents of BABT/SA salt segments on their mechanical properties was analyzed using tensile measurements. As shown in Figure 15B, PA6 has a tensile strength of 69.3 MPa. The addition of 10 wt % BABT/SA salt into the PA6 matrix increases the tensile strength of the copolymer (PA6-BABT/SA_10_) up to 105.5 MPa due to the high reinforcing ability of aromatic BABT/SA salt. The improvement in the ductility of copolyamides could be ascribed to the insertion of partially aromatic long chain comonomer into PA6 [46]. For PA6-BABT/SA copolyamides, the stress-strain curves showed a similar shape, and the tensile strength gradually dropped (105.2 to 46.6 MPa) with increasing weight content of BABT/SA salt segments. However, a superior strain at break was observed with the increased BABT/SA salt segments. The strains at break of PA6-BABT/SA copolyamides were 30.1%, 136.7% and 218.3% when the weight content of BABT/SA units was 10, 20 and 30 wt %, respectively. The strain at break of PA6-BABT/SA_30_ improved by 200% and the tensile strength dropped by about 36%, when compared to neat PA6, due to the random structure of PA6-BABT/SA_20_ and PA6-BABA/SA_30_ copolyamides. Inserting increasing weight content of BABT/SA segments randomly arranged the PA6 backbone. Therefore, the tensile strength gradually decreased. It is reasonable that the tensile strength would drop with the addition of above 10 wt % BABT/SA salt. Besides, the reduction in crystallinity and the change observed from the α phase to γ phase, in agreement with the DSC and XRD results, could explain the significant reduction in the tensile strength [41]. Nevertheless, loss in tensile strength is exchanged for a considerable increase in the strain at break. These results clearly show that PA6-BABT/SA copolyamides have outstanding mechanical properties compared to neat PA6 and commercially available PA66 [47].

### 3.8. Solubility of Resultant Polymers

The solubility of the polymers was determined by dissolving 100 mg of polymers in 10 mL of solvent at room temperature, and the results are listed in Table 6. The copolyamides exhibited good solubility in acidic solvents and insoluble in polar and less polar solvents at room temperature. Additionally, the copolyamides showed similar solubility as the neat PA6. These results indicate that the resultant copolyamides exhibit good solvent resistance.

### 3.9. Transparency of Copolyamides

**Figure 16:**
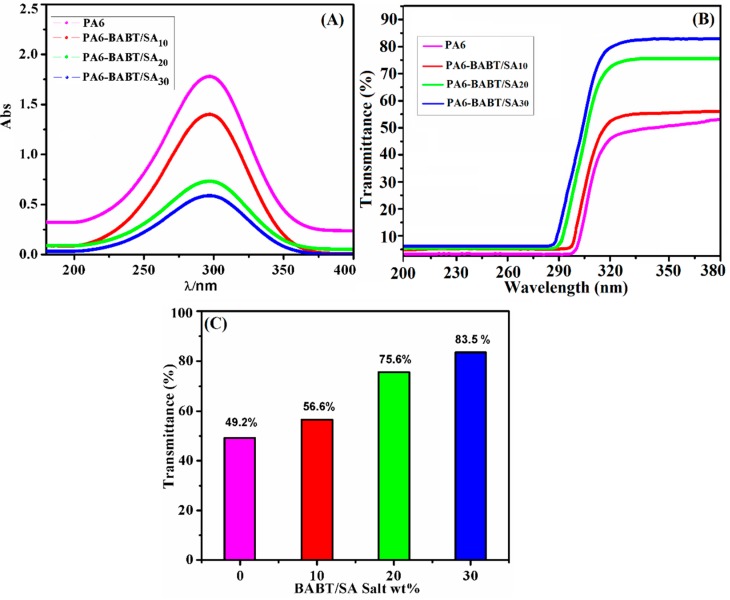
(**A**) The ultraviolet (UV) spectra of polyamide samples with different BABT/SA contents. (**B**) UV-visible transmittance spectra of neat PA6 and PA6-BABT/SA copolaymides. (**C**) Transmittance values of the polyamides films with different content of BABT/SA.

**Figure 17:**
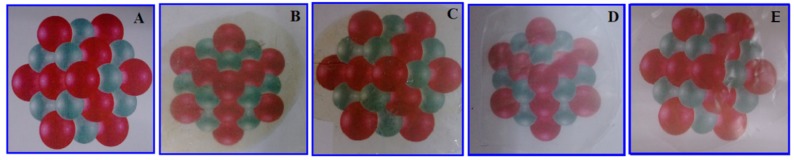
Transparency images of representative PA6 and PA6-BABT/SA copolyamides films: (**A**) original picture, (**B**) pure PA6, (**C**) PA6-BABT/SA_10_, (**D**) PA6-BABT/SA_20_, and (**E**) PA6-BABT/SA_30_.

The UV spectrum of PA6-BABT/SA copolyamides are shown in Figure 16A. The traces revealed that, along with the increased incorporation ratio of BABT/SA, both the intensity and area of the UV absorption peak decreased substantially. The transmittance value measured using a UV-transmission spectra is illustrated in Figure16B. The cut of the wave length values (λ_0_) of these copolymides were in the range of 280 to 310 nm. The transmittance of all the polyamide films was measured at 325 nm. The results shown in Figure 16C indicate that the transmittance of the copolymer films essentially continued to increase, and the maximum was 83.5% as the weight content of BABT/SA increased to 30 wt %. For all different ratios, the transparency of the as-synthesized copolymer was better than that of neat PA6. The traces are consistent with the XRD analysis and crystallinity data obtained from DSC of copolyamides. This also demonstrates that the transparency of the copolyamides could be improved considerably by adding 10 to 30 wt % BABT/SA segments. Specifically, as-synthesized copolyamides and the neat PA6, shaped to films with the same thickness of 30 μm, were stuck on a colored picture and photographed. The transparency images of representative PA6-BABT/SA copolymers and the reference PA6 are shown in Figure 17. Compared with the original picture shown in Figure 17A, all the copolymer films were colorless, whereas the reference film (PA6) was very slightly yellow, as illustrated in Figure 17B. This indicates that the PA6 homopolymer is easily oxidized at high temperatures. Additionally, as shown in Figure 17C, the polymer film had little wrinkles. When the content of BABT/SA salt increased from 10 to 30 wt %, the wrinkles disappeared and the copolymer films became more transparent [34].

## 4. Conclusions

Bio-based polyamides are engineering polymers based on renewable feedstocks that link green chemistry methods with superior thermal and mechanical properties. In this work and for the first time, we synthesized BABT diamine and BABT/SA aromatic polyamide salt, which can be produced from renewable resources. A class of bio-based copolyamides with variable physical and mechanical properties were synthesized from different weight contents of aliphatic/aromatic comonomer units via a melt condensation process. The chemical structures of the monomers and polymers were confirmed by FT-IR, ^1^H NMR, and ^13^C NMR spectroscopy. Depending on the BABT/SA salt content, the intrinsic viscosity and *M*_n_ of the copolyamides ranged from 0.94 to 1.33 dL/g and 23,600 to 37,100 Da, respectively. The aromatic BABT/SA salt disturbs the crystallization of the PA6 copolyamides and lowers the onset of *T*_m_ to contribute to the mobility of copolyamides and improve the application processability. The degree of crystallinity and *T*_g_ are also suppressed by increasing the BABT/SA salt weight ratio from 10 to 30 wt %. The copolyamide containing 30 wt % BABT/SA had a *T*_g_ of 32 °C, which is close to the human body temperature. The XRD analyses proved that pure PA6 exhibits the α-form. When BABA/SA salt reached 30 wt %, the copolyamides exhibited the γ-form. In addition, the novel copolyamides exhibited good thermal stability, similar to neat PA6. The tensile test showed that the incorporation of 10 wt % BABT/SA salt resulted in outstanding tensile strength (105.2 MPa) and elongation at break (30.1%), which are significant improvements over neat PA6. Further increasing the BABT/SA content from 20 to 30 wt %, the tensile strength substantially decreased and the elongation at break of the copolyamides gradually increased. Furthermore, the extremely diminished crystallinity of copolyamides endows them with good transparency. These results clearly showed that the synthesized polyamides could be useful for industrial application.

## Figures and Tables

**Table 1:** Carbon nuclear magnetic resonance (^13^C NMR) shift of corresponding peaks, shown in Figure 7.

**Carbon** **No.**	1	2	3	4	5	6	7	8	9
**Chemical shift**	5.3	24.5	24.7	27.9	42.3	43.1	25.2	29.8	41.6
**Carbon** **No.**	10	11	12	13	14	15	16	17	18
**Chemical shift**	34.3	24.9	25	25.1	125.2	128.7	179.8	180.1	173.2

**Table 2:** Composition and degree of randomness of the PA6-BABT/SA copolymers prepared by melt condensation reaction.

Samples	Composition Feed (PA6-BABT/SA)		Composition after MP by ^1^H NMR (PA6-BABT/SA)	Composition after MP by ^13^C NMR (PA6-BABT/SA)	Degree of Randomness
	wt %	mol %	mol %	mol %	R
PA6	100/0	100/0	-	-	-
PA6-BABT/SA_10_	90/10	97.6/2.4	98/2	N.A.	N.A.
PA6-BABT/SA_20_	80/20	94.7/5.3	95.2/4.8	94.9/5.1	1.05
PA6-BABT/SA_30_	70/30	91.2/8	92/8	91.8/8.2	1.03

MP: Melt Polymerization; N.A.: Not Available.

**Table 3:** Intrinsic viscosity and molecular weights of neat PA6 and PA6-BABT/SA copolymers.

Sample	Intrinsic Viscosity	*M*_n_	*M*_w_	*M*_p_	PDI
	dL/g	Da	Da	Da	
PA6	1.23	30,700	63,500	53,300	2.07
PA6-BABT/SA_10_	1.33	37,100	77,800	64,400	2.10
PA6-BABT/SA_20_	1.29	30,900	65,300	52,100	2.11
PA6-BABT/SA_30_	0.94	23,600	48,700	43,600	2.06

**Table 4:** Thermal properties and the calculated crystallization degrees of PA6 and PA6-BABA/SA copolyamides.

Sample	Onset (°C)	*T*_m_ (°C)	*T*_C_ (°C)	Δ*H*_m_ (J/g)	X_C%_	*T*_5%_ (°C)	*T*_max_ (°C)
BABT	172.4	217.1	-	140.1	-	194.25	203.3 and 447.7
BABT/SA	184.3	231.4	-	134.8	-	221.8	223.1 and 457.6
PA6	183.3	210.7	171.6	51.1	27.2	386.4	453.4
PA6-BABT/SA_10_	157	193.1	160.3	46.9	24.8	385.8	452.1
PA6-BABT/SA_20_	139.3	180.2	126.5	43.2	22.9	385.1	447.4
PA6-BABT/SA_30_	120	152.5	113.4	38.6	20.4	384.6	446.3

**Table 5:** Mechanical properties of PA6 and PA6-BABT/SA copolyamides.

Sample	*T*_g_ (°C)	Loss Modulus (E”)	Tensile Strength (MPa)	Elongation (%)
PA6	66.2	61.8	69.3	15.8
PA6-BABT/SA_10_	48.7	45.9	105.2	30.1
PA6-BABT/SA_20_	42.9	38.7	83.5	136.7
PA6-BABT/SA_30_	31.2	29.3	46 .6	218.3

**Table 6:** Solubility of PA6 and PA6-BABT/SA copolyamides.

Samples	THF	DMF	CHCl_3_	m-Cresol	DMAc	NMP	H_2_SO_4_	HCOOH	TFFA
PA6	-	-	-	-	-	-	++	++	++
PA6-PA6-BABT/SA_10_	-	-	-	-	-	-	++	++	++
PA6-BABT/SA_20_	-	-	-	-	-	-	++	++	++
PA6-BABT/SA_30_	-	-	-	-	-	-	++	++	++

**-**: insoluble; ++: soluble at room temperature. THF: tetrahydrofuran; DMF: *N*,*N*-dimethylformamide; CHCl_3_: chloroform; DMAc: *N*,*N*-dimethylacetamide; NMP: *N*-methyl-2-pyrrolidone; H_2_SO_4_: sulfuric acid; HCOOH: formic acid; CF_3_COOH: trifluoroacetic acid.
